# Curcumin Mitigates Fumonisin B_1_-Induced Ovarian Toxicity in Peak-Laying Ducks via Hormone Metabolic Protection and Enhanced Reproductive Resilience

**DOI:** 10.3390/toxins18010034

**Published:** 2026-01-09

**Authors:** Lihua Wang, Rui Liang, Qingyun Cao, Zhiwei Hou, Ali Mujtaba Shah, Qiuyi Deng, Xue Li, Jinze Li, Jiaqing Chen, Lukuyu A. Bernard, Muhammad Kashif Saleemi, Lin Yang, Wence Wang

**Affiliations:** 1 State Key Laboratory of Swine and Poultry Breeding Industry and Guangdong Provincial Key Laboratory of Animal Nutrition and Regulation, College of Animal Science, South China Agricultural University, Guangzhou 510642, China; 2 International Livestock Research Institute, Nairobi 00100, Kenya; 3 Department of Pathology, University of Agriculture Faisalabad, Faisalabad 38040, Pakistan; 4 Gurdon Institute and the Department of Genetics, University of Cambridge, Cambridge CB2 1QN, UK

**Keywords:** fumonisin B_1_, duck, curcumin, reproductive system

## Abstract

The objective of this study was to evaluate the protective effect of curcumin (Cur) on reproductive toxicity induced by fumonisin B_1_ (FB_1_) in laying ducks during the peak egg-laying period. A total of seventy-two 50-week-old Cherry Valley ducks were randomly assigned to four groups: control, FB_1_ (30 mg/kg), Cur (200 mg/kg), and Cur + FB_1_ (200 mg/kg + 30 mg/kg). The experiment lasted for 35 days. Our results showed that cur supplementation effectively restored the reductions in final body weight (*p* = 0.005) and oviduct length (*p* = 0.020) induced by FB_1_ exposure. Residual FB_1_ concentrations in serum, liver, and ovaries were markedly increased in the FB_1_-treated group, while Cur significantly decreased the FB_1_ residual in duck liver (*p* < 0.05). Meanwhile, Cur supplementation markedly counteracted the FB_1_-induced reductions in serum total protein, albumin, triglycerides, and high-density lipoprotein induced by FB_1_ exposure. Cur supplementation effectively regulated FB_1_-induced oxidative stress, inflammation, and endocrine disruption. Specifically, Cur lowered FB_1_-induced malondialdehyde levels (*p* < 0.010), attenuated interleukin-1β increase (*p* = 0.083), and reversed the reduction in immunoglobulin G levels. FB increased the levels of hormones associated with duck reproduction, including estradiol, follicle-stimulating hormone, and luteinizing hormone; in contrast, curcumin supplementation decreased the levels of these hormones (*p* < 0.010). Histopathological analysis revealed that Cur significantly alleviated the inflammation and necrosis in the liver, kidneys, ovaries, and oviducts induced by FB_1_. In conclusion, dietary Cur supplementation effectively alleviated FB_1_-induced reproductive toxicity in laying ducks by enhancing antioxidant capacity, improving lipid metabolism, and restoring hormonal homeostasis.

## 1. Introduction

Mycotoxin contamination has increasingly emerged as a critical constraint impairing quality and impeding sustainable development within the global feed and livestock production sectors. It poses a severe threat to the quality of livestock and poultry products, offspring development and human health, through impairment of the reproductive system and contamination of the food chain, respectively [1]. Fumonisins constitute a class of mycotoxins produced by specific species of Fusarium fungi, which are frequently detected in corn and other cereal grains, thereby exerting significant adverse health effects on both animals and humans [2]. Among fumonisins, fumonisin B_1_ (FB_1_), is the most prevalent and toxic congener, exhibiting primary toxicity toward the liver, kidneys, and reproductive organs in animals [3,4]. Notably, poultry exhibit heightened susceptibility to the toxicological effects of FB_1_ [5].

Studies have demonstrated that chickens exposed to FB_1_ through their diet experience growth retardation, tissue lesions, and disrupted intestinal microbiota [6]. Moreover, when laying hens are fed FB_1_-contaminated feed, both egg production and quality are significantly reduced [7]. FB_1_ disrupts the structural integrity and physiological function of reproductive organs. It reduces primary follicle count and increases the number of atretic follicles. Additionally, FB_1_ impairs hormone biosynthesis by inhibiting granulosa cell proliferation, thereby compromising the overall integrity of the reproductive system [8]. Prolonged exposure of pregnant animals to FB_1_ can trigger embryonic growth retardation and may further culminate in embryonic and fetal malformations or functional deficits [9]. Dietary administration of 10 mg/kg FB_1_ impairs egg-laying rate and egg weight in laying quails [10]. Additionally, FB_1_ has been found to reduce testicular weight, impair spermatogenesis, and significantly diminish semen quality in boars by reducing sperm viability and increasing sperm deformity rates [11].

Accumulating evidence demonstrates that plant-derived bioactive compounds, such as quercetin and resveratrol, protect against mycotoxin-induced toxicity [12,13]. Notably, curcumin (Cur), a polyphenolic compound from the Zingiberaceae plant *Curcuma longa* L., exerts pleiotropic effects on various molecular targets and key signaling pathways [14]. Recent studies have demonstrated that Cur can effectively counteract the toxic effects induced by various mycotoxins. Specifically, it had been proved that Cur can effectively alleviate the adverse effects caused by FB_1_ through the modulation of IRE1/MKK7/JNK/Caspase3 pathway [15,16]. Curcumin alleviates Aflatoxin B_1_-induced hepatic toxicity in ducks by inhibiting endoplasmic reticulum stress and restoring lipid metabolism balance [17]. An in vivo study also showed that Cur alleviated Aflatoxin B_1_-induced renal toxicity in ducks by inhibiting mitochondrial-mediated oxidative stress and regulating abnormal iron phagocytosis and exocytosis [18]. Additionally, dietary supplementation with Cur has been reported to mitigate adverse physiological outcomes in intrauterine growth retardation (IUGR) weaned piglets, which effectively reduces lipid oxidation, lowers plasma inflammatory factor levels, and enhances antioxidant capacity [19]. Although Cur has been shown to protect hepatic and renal function, its protective effects and underlying mechanisms against FB_1_-induced reproductive toxicity remain inadequately understood.

As curcumin has proven multiple effects in alleviating oxidative stress and protecting mitochondrial function, we hypothesize that it may also mitigate the reproductive impairment in breeder ducks caused by FB_1_. Therefore, we conducted this study with the aim of evaluating the potential therapeutic effects of Cur on FB_1_-induced reproductive impairment in ducks with serum biocharacters, antioxidants, inflammatory, and immune indicators, and hormone levels.

## 2. Results

### 2.1. Determination of the Content of Fermentation Product FB_1_

The FB_1_ content of the fermentation product was determined, and its concentration was 16.235 mg/g (Table 1).

### 2.2. The Effect of FB_1_ and Cur on the Growth Performance in Peak-Laying Ducks

Dietary exposure to FB_1_ resulted in a significant reduction in the final weight of laying ducks, whereas Cur supplementation mitigated this FB_1_-induced decrease (*p* = 0.005, Table 2). No significant differences were observed in feed intake or average egg production among the four experimental groups.

### 2.3. FB_1_ Residues of FB_1_ Exposure and Cur Treatment Ducks

**Figure 1:**
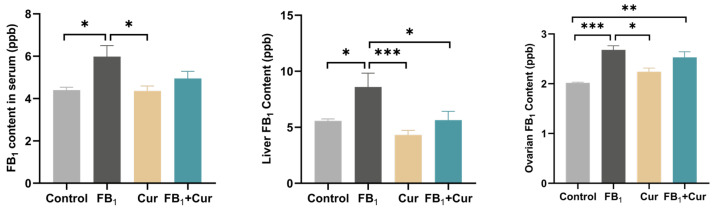
FB_1_ residues of FB_1_ exposure and Cur treatment in peak-laying ducks. FB_1_ = Fumonisin B_1_; Cur = curcumin. *p* < 0.05 is represented by *, *p* < 0.01 by **, *p* < 0.001 by ***.

The results showed that Cur supplementation significantly reduced the FB_1_-induced elevation of FB_1_ levels in the liver of laying ducks (*p* < 0.050). However, it had no significant effect on the FB_1_-induced increase in the blood and ovaries (Figure 1).

### 2.4. The Effect of FB_1_ and Cur on Organ Parameters in Peak-Laying Ducks

Experimental findings indicated no significant differences in organ indices between FB_1_-exposed laying ducks and the control group (Table 3). However, Cur supplementation significantly increased oviduct length (*p* = 0.020) and reduced the relative weight of the oviduct isthmus (*p* = 0.0003) compared to the FB_1_-exposed group. Additionally, FB_1_ exposure showed a trend toward increased ovarian relative weight (*p* = 0.074) (Table 4).

### 2.5. The Effect of FB_1_ and Cur on Serum Biochemical Indices in Peak-Laying Ducks

**Figure 2:**
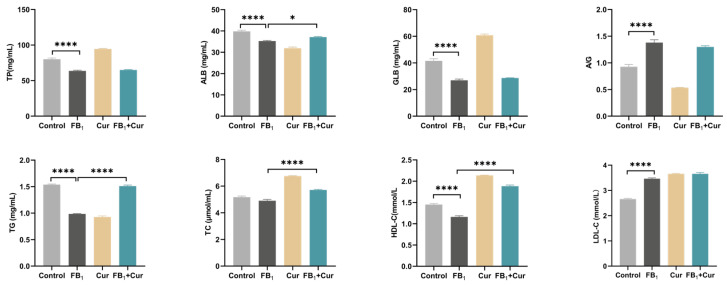
Blood biochemical indices of FB_1_ exposure and Cur treatment in peak-laying ducks. TP = total protein; ALB = albumin; GLB = globulin; A/G = albumin/globulin; TG = triglyceride; TC = total cholesterol; HDL-C = high-density lipoprotein cholesterol; LDL-C = low-density lipoprotein cholesterol., *p* < 0.05 is represented by *, *p* < 0.0001 by ****.

Dietary FB_1_ exposure significantly decreased serum total protein (TP), albumin (ALB), and globulin (GLB) levels, while increasing the A/G ration. In contrast, Cur supplementation significantly increased serum ALB levels. Results showed that dietary FB_1_ exposure significantly decreased triglyceride (TG) and high-density lipoprotein cholesterol (HDL-C) levels, while increased low-density lipoprotein cholesterol (LDL-C) levels. Cur supplementation attenuated these FB_1_-induced alterations by inhibiting the decline in TG and HDL-C levels. Additionally, Cur supplementation increased serum TC levels (Figure 2).

### 2.6. The Effect of FB_1_ and Cur on Antioxidant Indices in Peak-Laying Ducks

**Figure 3:**
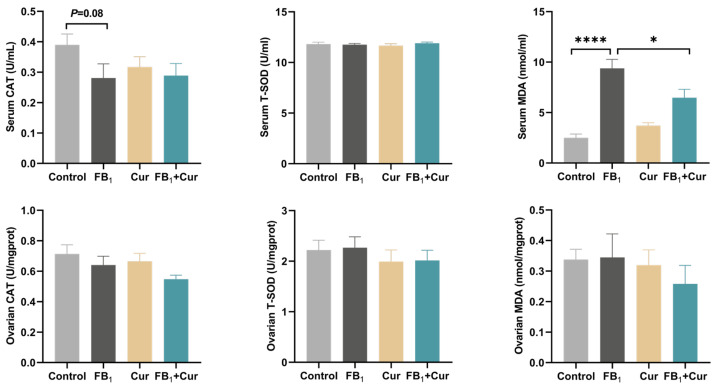
Serum and ovarian antioxidant indices of FB_1_ exposure and Cur treatment in peak-laying ducks. CAT = catalase; T-SOD = Total superoxide dismutase; MDA = malondialdehyde. *p* < 0.05 is represented by *, *p* < 0.0001 by ****.

FB_1_ exposure tended to decrease catalase (CAT) activity (*p* = 0.08) in duck serum and increase malondialdehyde (MDA) levels (*p* < 0.0001), a biomarker of lipid peroxidation. Notably, Cur supplementation mitigated the FB_1_-induced elevation of MDA levels (*p* < 0.05, Figure 3). In contrast, neither FB_1_ exposure nor Cur supplementation significantly affected the antioxidant indices in the ovary.

### 2.7. The Effect of FB_1_ and Cur on Sex Hormone Indices in Peak-Laying Ducks

**Figure 4:**
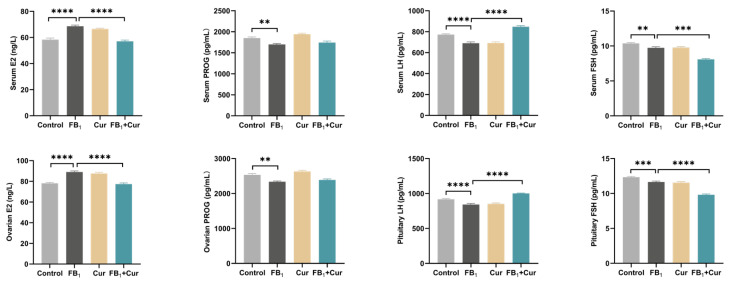
Hormonal indices of FB_1_ exposure and Cur treatment in peak-laying ducks. E2 = estradiol; PROG = progesterone; LH = luteinizing hormone; FSH = follicle-stimulating hormone. *p* < 0.01 is represented by **, *p* < 0.001 by ***, *p* < 0.0001 by ****.

Results showed that Cur supplementation attenuated the FB_1_-induced elevation of estradiol (E2) levels in serum and ovaries and reversed the FB_1_-induced reduction in luteinizing hormone (LH) levels in serum and pituitary glands. However, Cur supplementation had no significant effect on the FB_1_-induced reduction in progesterone (PROG) levels in serum and ovaries, nor on the reduction in follicle-stimulating hormone (FSH) levels in serum and pituitary glands (Figure 4).

### 2.8. The Effect of FB_1_ and Cur on Immune and Inflammatory Indices in Peak-Laying Ducks

**Figure 5:**
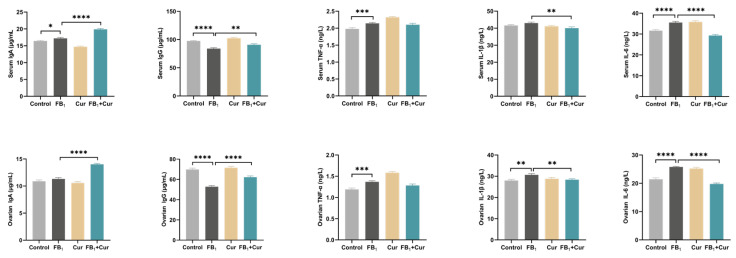
Immune and inflammatory indices of FB_1_ exposure and Cur treatment in peak-laying ducks. IgA = immune globulin A; IgG = immune globulin G; TNF-α = tumor necrosis factor; IL-1β = Interleukin-1β; IL-6 = Interleukin-6. *p* < 0.05 is represented by *, *p* < 0.01 by **, *p* < 0.001 by ***, *p* < 0.0001 by ****.

This study found that dietary Cur supplementation reversed the FB_1_-induced reduction in immunoglobulin G (IgG) levels and attenuated the FB_1_-induced elevation of the interleukin-1β (IL-1β) and interleukin-6 (IL-6). However, Cur supplementation had no significant effect on the FB_1_-induced increase in tumor necrosis factor-α (TNF-α) levels. Additionally, Cur supplementation significantly increased immunoglobulin A (IgA) levels (Figure 5).

### 2.9. The Effect of FB_1_ and Cur on Pathological Sections in Peak-Laying Ducks

**Figure 6:**
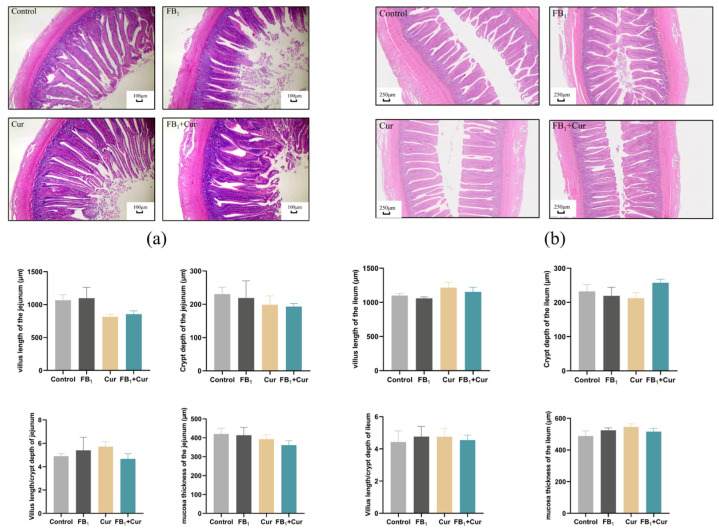
Intestinal morphology of FB_1_ exposure and Cur treatment in peak-laying ducks. (**a**) Pathological section of the jejunum, magnification, 4×; scale bar, 100 μm. (**b**) Pathological section of the ileum, magnification, 4×; scale bar, 250 μm.

**Figure 7:**
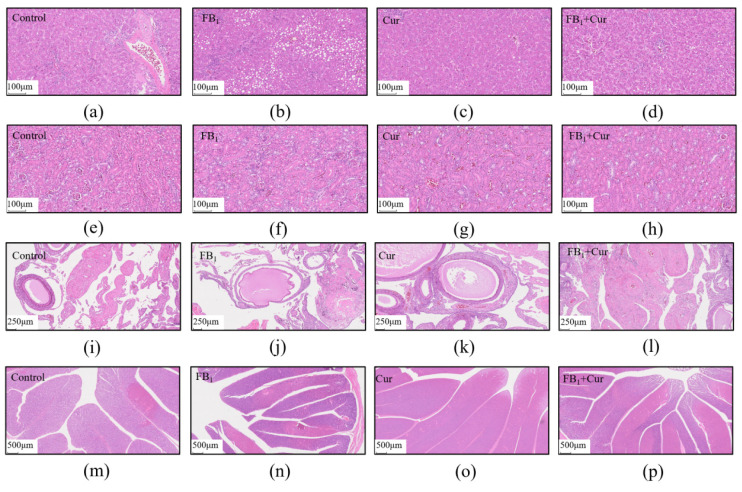
Pathological damage of FB_1_ exposure and Cur treatment in peak-laying ducks. (**a**–**d**) Liver pathological section, magnification, 10×; scale bar, 100 μm. (**e**–**h**) Kidney pathological section, magnification, 10×; scale bar, 100 μm. (**i**–**l**) Ovarian pathological section, magnification, 10×; scale bar, 250 μm. (**m**–**p**) Oviducts pathological section, magnification, 4×; scale bar, 500 μm.

Dietary FB_1_ exposure and Cur supplementation did not significantly impact the intestinal development of the adult ducks (Figure 6), including jejunal and ileal villus length, crypt depth, and muscle thickness. However, FB_1_ caused hepatic steatosis in ducks, as evidenced by the presence of lipid vacuoles of varying sizes in hepatocyte cytoplasm in hepatic tissue sections (Figure 7a–d). Conversely, Cur supplementation significantly ameliorated the occurrence of these hepatic lipid vacuoles. No significant differences were observed in kidney tissue sections (Figure 7e–h).

Ovarian histopathological sections revealed that Cur supplementation could alleviate the separation of the follicular granulosa layer from the follicular membrane, as well as the concurrent localized fibrotic lesions in the FB_1_ group (Figure 7i–l). Histopathological examination of the oviducts demonstrated that FB_1_ induced partial epithelial detachment and the formation of hemorrhagic foci. In contrast, Cur effectively inhibited epithelial detachment and the development of additional pathological lesions (Figure 7m–p).

## 3. Discussion

Mycotoxin contamination is a widespread and critical issue in animal husbandry, often impairing reproductive performance in livestock and poultry. Using plant extracts to mitigate the adverse effects of mycotoxins in feed is a promising and sustainable strategy. In this study, we systematically evaluated the effects of Cur on growth performance, organ development, serum biochemical parameters, histopathological changes, antioxidant capacity, hormone levels, and immune-inflammatory responses in peak-laying Cherry Valley ducks exposed to FB_1_. FB_1_ exposure significantly reduced the final body weight of laying ducks. FB_1_ disrupts sphingolipid metabolism by inhibiting sphingosine synthase activity, suppressing cell proliferation and inducing apoptosis, leading to growth retardation and histopathological damage [20,21,22]. Consistent with these findings, Butkeraitis et al. reported that FB_1_ significantly decreased feed intake in laying quails, reducing weight gain [23]. Studies also show that FB_1_ can delay early embryonic development in ducks by inhibiting ceramide synthases and folate transporters, disrupting the sphingolipid metabolic pathway [24]. In our study, Cur promoted growth by improving lipid metabolism and regulating hormone levels. Previous studies research suggests that Cur may counteract FB_1_-induced growth inhibition through two mechanisms: (1) activation of the AMPK/mTOR signaling pathway to enhance protein synthesis, and (2) suppression of pro-inflammatory cytokines (e.g., TNF-α) to reduce energy expenditure [25]. Furthermore, Cur has been shown to improve production performance, antioxidant enzyme activity, and immune function in laying hens under high-temperature stress by modulating lipid metabolic pathways [26].

FB_1_ is difficult to eliminate metabolically due to its stable structure and lack of recognition sites for metabolic enzymes, resulting in its accumulation in poultry tissues and organs [27]. The efficiency of toxin transformation and residual deposition depends on the poultry’s health and liver biotransformation capacity [28]. This study shows that FB_1_ primarily accumulates in the liver when transported through the circulatory system, with lower deposition in the ovaries. A study on 21-day-old chickens fed 20 mg/kg diet of FB_1_ + FB_2_ for 4 and 9 days found that FB_1_ accumulation in the liver significantly increased, with concentrations at 20.3 and 32.1 ng/g, respectively [29]. After 12 days of feeding 85-day-old male mule ducks with FB_1_, the residual FB_1_ levels in their livers were significantly higher compared to the control group [30]. These results suggest that prolonged exposure leads to FB_1_ accumulation in the liver. Furthermore, feeding 10 mg/kg of FB_1_ to 10-day-old broilers for 21 days raised FB_1_ residues in the gizzard [31]. Cur, an effective detoxifying substance, has been shown to reduce AFB_1_ residues in the liver and muscles of broilers [32]. This study also indicates that Cur can decrease FB_1_ accumulation in the liver. Curcumin, a polyphenolic compound, forms hydrophobic interactions with FB_1_’s long-chain carboxylic acid and hydroxyl groups, disrupting its structure [33,34]. Additionally, Curcumin upregulates the expression and activity of liver CYP450 enzymes and glucuronosyltransferase, which hydroxylate and glucuronidate FB_1_, increasing its water solubility and facilitating its excretion via urine or bile [35,36].

Ovary structure, follicle count, and fallopian tube development are closely linked to production performance. In this study, feeding laying hens with FB1 tended to lead to an increase in ovary weight. Similarly, after 4 weeks of FB_1_ feeding, female rats exhibited increased ovary weight and decreased follicle number, impairing reproductive function [37]. The observed phenomena in the FB_1_-fed hens may be due to the separation of the granular and capsule layers in the ovary, along with local fibrotic lesions. Additionally, elevated E2 levels promote ovarian stromal cells proliferation, while insufficient LH levels hinder ovulation, exacerbating follicular retention and increasing ovary weight [38]. These findings align with this study, where FB_1_ significantly elevated E2 levels in serum and ovaries while reducing LH levels. Cur has been shown to regulate both hormones, impairing the release of mature follicles and promoting the retention of immature follicles, disrupting follicle development homeostasis and leading to a slight increase in ovary weight. Notably, Cur supplementation alleviates these pathological changes, not by altering weight, but through hormonal regulation and pathological improvement. This suggests its potential to mitigate organ dysfunction caused by mycotoxins.

Data indicate that an FB_1_ level of 32 mg/kg in the diet increases total cholesterol (TC) and LDL levels in duck serum, and at 128 mg/kg, it raises TP content. These findings are consistent with our results, where FB_1_ in feed decreases serum TP levels and increases LDL-C levels [39]. Tardieu et al. also demonstrated that FB_1_ elevates LDL levels in turkey serum [40]. Additionally, this study observed a decrease in ALB, TG, and HDL-C levels, confirming FB_1_’s toxic effect on liver synthesis and lipid metabolism. FB_1_ disrupts lipoprotein metabolism and reverses cholesterol transport via the sphingolipid signaling pathway [41]. Cur supplementation significantly increases serum ALB levels, alleviates the reduction in TG and HDL-C, and raises TC. Similarly, Cur has been shown to reduce TG, TC, and LDL-C levels in the serum of laying quails [42], and Kong et al. research indicates that Cur supplementation decreases TC, TG, and AST levels in laying hens [43]. These effects are attributed to Cur promoting the transfer of cholesterol from cells to HDL particles, enhancing fatty acid oxidation, reducing lipid accumulation, and alleviating lipid metabolism disorders caused by FB_1_ [44,45].

FB_1_ can cause diffuse vacuolation and focal mononuclear cell infiltration in the liver of laying hens [46]. Similarly, when different doses of FB_1_ (0–4.374 mg/kg BW) were administered to mice for 8 weeks, liver tissue exhibited pathological changes, including necrotic inflammation, vacuolar degeneration, and fragmented necrosis [47]. FB_1_ exposure also disrupts the homeostasis of the liver cytochrome P450 system and activates endoplasmic reticulum stress, leading to liver damage [48]. Cur alleviates liver steatosis caused by FB_1_ by reducing residual FB_1_ in the liver. Although Cur does not significantly reduce FB_1_ residues in serum and ovaries, it mitigates granulosa cell shedding and the shedding of oviduct epithelium These findings suggest that Cur exerts its protective effects not by enhancing FB_1_ metabolism or excretion, but by directly interfering with its toxic signaling pathways such as stress and lipid metabolism. Studies show that curcumin can inhibit oxidative stress and AFB_1_-induced liver damage in ducks [49]. In line with this, Chen et al. demonstrated that Cur inhibits the endoplasmic reticulum stress pathway activated by FB_1_, reducing apoptosis in PK-15 cells, further supporting this hypothesis [15]. FB_1_ primarily damages ovarian structure and function, leading to a decrease in primary follicles, an increase in degenerated follicles, and impaired hormone synthesis due to inhibited granulosa cell proliferation [50]. This study found that FB_1_ not only reduced E2 levels and increased LH levels but also decreased PROG and FSH levels. However, Cur did not regulate this pathway. During the peak laying period, FSH, LH, and progesterone secretion levels gradually increased [51,52]. We hypothesize that FB_1_ damages the ovary, delaying or blocking follicle development and reducing progesterone synthesis [53]. It may also disrupt the hypothalamic-pituitary-gonadal axis, leading to decreased FSH secretion and affecting reproductive function in laying ducks [54].

Exposure to FB_1_ reduces serum CAT activity and increases MDA levels, highlighting oxidative stress as a key toxic mechanism. Supplementation with 200 mg/kg Cur in laying hens decreases liver TG content and MDA levels, suggesting that Cur improves lipid metabolism and oxidative status in the liver [55]. Cur also protects the ileum of ducks from AFB_1_-induced damage and oxidative stress by reducing plasma AFB_1_-DNA adducts [56]. Furthermore, dietary Cur supplementation mitigates H_2_O_2_-induced oxidative damage and reproductive decline in roosters [57]. Its antioxidant activity, attributed to phenolic hydroxyl groups, helps neutralize free radicals and scavenge reactive oxygen species (ROS) [58]. These findings indicate that curcumin, as an antioxidant, alleviates stress and enhances poultry health. Additionally, FB_1_ exposure significantly elevates TNF-α and IL-1β levels in serum and ovaries, contributing to intestinal inflammation in broilers [59,60]. Cur inhibits the secretion of these pro-inflammatory cytokines, aligning with Li et al.’s results, which show that Cur alleviates AFB_1_-induced liver damage in chickens by regulating pro-inflammatory factors (TNF-α, iNOS, IL-6, and IL-1β) [61]. Cur exerts anti-inflammatory effects by inhibiting inflammatory signaling pathways, demonstrating its ability to alleviate FB_1_-induced tissue damage through a synergistic “antioxidant-anti-inflammatory” effect [62].

## 4. Conclusions

Dietary FB_1_ exposure results in toxin residues in the tissues and organs of peak-laying ducks, which induce body weight reduction and dysregulation of hormone levels and inflammatory factors, ultimately contributing to hepatic and ovarian damage. In contrast, Cur supplementation effectively mitigates FB_1_-induced toxicity by improving body weight, restoring hormone homeostasis in peak-laying ducks. Our study provides fundamental research This study provides for the application of plant extracts in alleviating mycotoxin-induced damage in breeder ducks.

## 5. Materials and Methods

This study was conducted at the Teaching and Research Base of South China Agricultural University, Guangzhou, China. This experiment was approved by the Animal Protection and Use Committee of South China Agricultural University (Approval No.: 2025G007).

### 5.1. Animals, Diets and Experimental Treatments

A total of seventy-two peak-laying Cherry Valley ducks, with an average body weight of 3.23 ± 0.345 kg and 50 weeks of age, were selected based on similar health status and randomly assigned to four experimental groups: Control, FB_1_ (30 mg/kg diet), Cur (200 mg/kg diet), and FB_1_ + Cur (30 mg/kg diet FB_1_ + 200 mg/kg diet Cur). Each group consisted of six replicates, with three ducks per replicate housed in individual cage. The feeding trial lasted for 42 days, including a 7-day pre-feeding period and a 35-day experimental feeding period.

The FB_1_ used in this study was produced by fermenting rice with *Fusarium verticillioides* at 25 °C for 28 days under light-protected conditions. The FB_1_ content was quantified using high-performance liquid chromatography–tandem mass spectrometry (HPLC-MS/MS) [63], and the FB_1_ supplementation level adhered to dietary hygiene standards (≤60 mg/kg) (NY/T 1970). Cur was purchased from Macklin Biochemical Technology Co., Ltd. (Shanghai, China). Both FB_1_ and Cur were incorporated into the basal diet according to the experimental design, with the diet formulation provided in Table 5. Throughout the experiment, all ducks had ad libitum access to feed and water. Feed was withdrawn at 22:00 in the evening prior to trial termination. Lighting was provided from 06:00 to 22:00 daily, and the health status of the ducks was monitored daily to ensure their well-being.

### 5.2. FB_1_ Fermentation, Extraction, and Content Determination in Feeds

A 250 mL conical flask was used, into which 50 g of rice and 20 mL of ultrapure water were added. The rice was soaked overnight, followed by autoclaving at 121 °C for 1 h for sterilization. After cooling in a laminar flow hood, the rice mass was loosened with a glass rod, and fungal mycelium plugs from potato dextrose agar (PDA) medium were inoculated into the rice. The plugs were thoroughly mixed to ensure complete contact between the mycelium and the medium. The inoculated rice was then cultured under dark conditions at 25 °C for 28 days, with daily manual agitation during the initial cultivation phase to maintain continuous contact between the mycelium and the rice. After cultivation, the rice was harvested, dried at 55 °C, and sieved through a 40-mesh sieve. The dried rice was stored at −20 °C for future use.

A 1.0 ± 0.01 g aliquot of the pulverized rice was accurately weighed into a 15 mL centrifuge tube, and 7.5 mL of acetonitrile-water (50:50, *v*/*v*) was added as the extraction solvent. The mixture was vortexed for 10 min then centrifuged at 3000 rpm for 5 min. The supernatant was collected, and the extraction process was repeated once. The two supernatants were combined and mixed to a final volume of 14 mL. A 2 mL aliquot of the combined supernatant was transferred to a fumonisin purification tube (SBEQ-CA8805-H, CNW QuEChERS Custom Purification Tubes containing 200 mg MgSO_4_, 100 mg Sodium citrate, 100 mg NaCl, and 100 mg C_18_). The mixture was vortexed and shaken for 2 min to ensure complete mixing. The fumonisin purification tubes were obtained from Anpel Laboratory Technologies (Shanghai, China). The supernatant was then centrifuged at 3000 rpm for 5 min, and the resulting supernatant was dried under nitrogen at 40 °C using a nitrogen evaporator. After re-solubilization with the extractant solvent, the sample was used for FB_1_ content determination. The HPLC-MS/MS operation parameters were as follows: Chromatographic Conditions: (Mobile Phase A: 1 mL formic acid and 1 mL of a 100 mmol/L ammonium acetate solution, diluted to 1 L with water; Mobile Phase B: 900 mL methanol mixed with 1 mL formic acid, then diluted to 1 L with water; Column: C18 reversed-phase liquid chromatography column; Column temperature: 40 °C; Flow rate: 0.3 mL/min; Injection volume: 1 µL; Elution Program: Gradient elution was performed as outlined in Table 6). Mass Spectrometric Conditions: (Ionization: Electrospray ionization (ESI) in both positive (ESI+) and negative (ESI−) ion modes; Detection: Multiple reaction monitoring (MRM); Capillary voltages: 0.6 kV (ESI+) and 2.5 kV (ESI−); Ion source temperature: 150 °C; Desolvation temperature: 500 °C; Nitrogen flow rate: 1000 L/h).

### 5.3. Growth and Production Performance

The body weight of all ducks was recorded before and after the experimental trial. Daily feed intake was monitored by weighing the provided feed, and residual feed was weighed weekly. Eggs were collected daily starting at 06:00, with the final collection occurring at 09:00 on the last day of the trial. The number of eggs laid per replicate pen was recorded.

### 5.4. Blood Characteristics

On day 36 of the experiment, 12 ducks with average body weight were selected from each treatment group. Blood samples were collected via jugular vein puncture into tubes and centrifuged at 3000× *g* for 15 min to obtain serum. The serum was aliquoted into 1.5 mL microcentrifuge tubes and stored at −20 °C until biochemical analysis. Serum concentrations of TP, ALB, GLB, TC, TG, LDL-C, and HDL-C were measured using an automatic biochemical analyzer (Guangzhou Daan Gene Biotechnology Co., Ltd., Guangzhou, China).

### 5.5. Relative Organ Index

After serum collection, the ducks were euthanized by carbon dioxide inhalation and cervical dislocation, performed by trained personnel. The weights of the liver, kidney, spleen, and pancreas were recorded. Reproductive organs were dissected, and their lengths were measured. For ovaries and oviducts, the weights of the whole organs as well as the dilated segment, isthmus, and uterine region were recorded.

Relative weight = (Organ weight)/(Final BW) × 100.

Relative length = (Organ length)/(Full Length) × 100.

### 5.6. Antioxidative Assays

Ovarian, and pituitary tissues were isolated, placed into cryopreservation tubes, snap-frozen in liquid nitrogen, and stored at −80 °C for later analysis. Antioxidant parameters, including CAT (A007-1), total superoxide dismutase (T-SOD, A001-3), and MDA (A003-1) were assayed using kits from Nanjing Jiancheng Bioengineering Institute (Nanjing, China).

### 5.7. ELISA Kit Detection Indicators

Immune indicators (IgG, IgA), inflammatory cytokines (TNF-α, IL-1β, IL-6), sex hormones (FSH (13649), LH (13645), E2 (14364), PROG (13643)) were quantified using enzyme-linked immunosorbent assay (ELISA) kits (Yancheng, China). All assays were performed following the manufacturers’ instructions. FB_1_ residues in the serum, liver, and ovarian tissues were determined using an ELISA kit (Shanghai Hengyuan Biotechnology Co., Ltd., Shanghai, China) according to the manufacturer’s instructions.

### 5.8. H&E Staining

Liver, kidneys, ovaries, and intestinal samples (1 cm from the mid-jejunum and mid-ileum) were collected, rinsed three times with phosphate-buffered saline (PBS), and fixed in 4% formaldehyde solution. The fixed samples were processed through a graded ethanol dehydration series, paraffin embedding, sectioning, and mounting on glass slides. The sections were dewaxed in xylene, rehydrated through ethanol, stained with hematoxylin and eosin (H&E), and mounted with neutral balsam for histological examination [64].

### 5.9. Statistical Analysis

Data were collected and analysis using *t*-test in GraphPad Prism 9.4.1 (GraphPad Software, San Diego, CA, USA). Differences between treatments were evaluated using the *t*-test. Statistical significance was set at *p* ≤ 0.05, with * indicating *p* < 0.05, ** indicating *p* < 0.01, *** indicating *p* < 0.001, and **** indicating *p* < 0.0001. Measurements of intestinal villus length, crypt depth, and muscularis propria thickness were performed using ImageJ software (v 1.8.0, National Institutes of Health, Bethesda, MD, USA).

## Figures and Tables

**Table 1:** The content of the fermentation product FB_1_ (mg/g).

Item	Content
FB_1_	16.235 ± 0.006

**Table 2:** Growth performance of FB_1_ exposure and Cur treatment in peak-laying ducks.

Item	Treatment				SEM	*p*-Value
	Control	FB_1_	Cur	FB_1_ + Cur		
Initial weight (kg)	3.236	3.297	3.219	3.322	0.024	0.335
Final weight (kg)	3.289 ^a^	2.993 ^b^	3.139 ^ab^	3.200 ^a^	0.062	0.005
Feed intake (g/day)	209.590	210.666	203.047	213.194	2.162	0.648
Average egg production	5.520	5.547	5.480	6.160	0.162	0.423

FB_1_ = Fumonisin B_1_; Cur = curcumin. ^a,b^ Different superscripts within a row indicate significant differences at *p* ≤ 0.05.

**Table 3:** Organ indices of FB_1_ exposure and Cur treatment in peak-laying ducks (g/kg).

Item	Treatment				SEM	*p*-Value
	Control	FB_1_	Cur	FB_1_ + Cur		
liver	16.503	16.462	17.044	15.731	0.269	0.732
kidney	6.336 ^ab^	5.856 ^b^	6.596 ^a^	5.886 ^b^	0.180	0.006
spleen	0.612	0.486	0.627	0.545	0.032	0.435
pancreas	2.227	2.113	2.126	2.219	0.030	0.759

FB_1_ = Fumonisin B_1_; Cur = curcumin. ^a,b^ Different superscripts within a row indicate significant differences at *p* ≤ 0.05.

**Table 4:** Reproduction organ indices of FB_1_ exposure and Cur treatment in peak-laying ducks.

Item	Treatment				SEM	*p*-Value
	Control	FB_1_	Cur	FB_1_ + Cur		
Ovary (g/kg)	2.066	2.276	2.042	2.316	0.070	0.074
Oviductal length (cm/cm)	20.967 ^ab^	20.696 ^b^	21.934 ^ab^	22.909 ^a^	0.503	0.020
Relative length (cm/cm)						
Oviductal bulge	9.586	8.892	9.718	9.653	0.192	0.251
Oviductal isthmus	2.710	2.934	2.725	2.573	0.075	0.128
Oviductal uterine segment	6.472	6.757	6.611	6.751	0.068	0.892
Relative weights (g/kg)						
Oviductal bulge	0.651	0.647	0.657	0.649	0.002	0.795
Oviductal isthmus	0.234 ^ab^	0.246 ^a^	0.204 ^c^	0.220 ^bc^	0.009	0.000
Oviductal uterine segment	0.114	0.113	0.107	0.119	0.002	0.195

FB_1_ = Fumonisin B_1_; Cur = curcumin. ^a–c^ Different superscripts within a row indicate significant differences at *p* ≤ 0.05.

**Table 5:** Feed formulation and nutritional composition.

Ingredient	Content (100%)
Corn	52.550
Soybean meal	35.860
Soybean oil	1.600
Stone powder	7.380
Calcium hydrogen phosphate	1.920
NaCl	0.260
D, L-Methionine	0.170
L-Lysine hydrochloride	0.100
L-Threonine	0.010
Multivitamin ^1^	0.050
Multimineral ^2^	0.100
Nutrients ^3^	
Crude protein	19.26
Crude fiber	5.17
Crude ash	11.88
Calcium	3.65
Phosphorus	0.67

^1^ Product analysis Guaranteed value of 1 kg premix: vitamin A 4 × 10^7^ IU, Vitamin D_3_ 1 × 10^7^ IU, Vitamin E 1 × 10^5^ mg, Vitamin K 3.2 × 10^5^ mg, Vitamin B 1.1 × 10^5^ mg, Vitamin B_2_ 30,000 mg, Vitamin B_6_ 20,000 mg, Vitamin B_12_ 100 mg, biotin 500 g, D-pantopanic acid 60,000 mg, folic acid 5000 mg, nicotinamide 2 × 10^5^ mg, ethoxyquinoline 500 mg. ^2^ Guaranteed value of trace element analysis for poultry products: Fe^2+^ 100–110 g/kg, Cu 8–12 g/kg, Mn 120–130 g/kg, Co 0.4–0.6 g/kg, Se 0.3–0.5 g/kg, I 0.7–0.9 g/kg. ^3^ is the measured value.

**Table 6:** Mobile phase gradient elution procedure.

Time (min)	A (%)	B (%)
0	95	5
2	95	5
4	80	20
12	5	95
12.1	1	99
13	1	99
13.5	95	5
16	95	5

## Data Availability

The original contributions presented in this study are included in the article. Further inquiries can be directed to the corresponding authors.
